# Hereditary C1q Deficiency is Associated with Type 1 Interferon-Pathway Activation and a High Risk of Central Nervous System Inflammation

**DOI:** 10.1007/s10875-024-01788-5

**Published:** 2024-08-28

**Authors:** Clément Triaille, Neha Mohan Rao, Gillian I. Rice, Luis Seabra, Fraser J. H. Sutherland, Vincent Bondet, Darragh Duffy, Andrew R. Gennery, Benjamin Fournier, Brigitte Bader-Meunier, Christopher Troedson, Gavin Cleary, Helena Buso, Jacqueline Dalby-Payne, Prajakta Ranade, Katrien Jansen, Lien De Somer, Marie-Louise Frémond, Pallavi Pimpale Chavan, Melanie Wong, Russell C. Dale, Carine Wouters, Pierre Quartier, Raju Khubchandani, Yanick J. Crow

**Affiliations:** 1 Division of Pediatric Rheumatology, Department of Pediatrics, University Hospitals Leuven, Leuven, Belgium; 2 Pôle de Pathologies Rhumatismales Systémiques Et Inflammatoires, Institut de Recherche Expérimentale Et Clinique, Université Catholique de Louvain, Brussels, Belgium; 3 Department of Pediatric Rheumatology, NH SRCC Hospital, Mumbai, Maharashtra India; 4 Division of Evolution and Genomic Sciences, School of Biological Sciences, Faculty of Biology, Medicine and Health, University of Manchester, Manchester Academic Health Science Centre, Manchester, UK; 5 Laboratory of Neurogenetics and Neuroinflammation, Imagine Institute, INSERM UMR1163, Paris, France; 6 MRC Human Genetics Unit, Institute of Genetics and Cancer, University of Edinburgh, Edinburgh, UK; 7 Translational Immunology Unit, Institut Pasteur, Université Paris-Cité, Paris, France; 8 Translational and Clinical Research Institute, Newcastle University, Newcastle Upon Tyne, UK; 9 Paediatric Stem Cell Transplant Unit, Great North Children’s Hospital, Newcastle Upon Tyne, UK; 10 Paediatric Immunology-Hematology and Rheumatology Unit, Necker Hospital, APHP Centre, Université Paris-Cité, Paris, France; 11 T. Y. Nelson Department of Neurology and Neurosurgery, Children’s Hospital at Westmead, University of Sydney, Westmead, NSW Australia; 12 Paediatric Rheumatology, Alder Hey Children’s Hospital, Liverpool, UK; 13 Department of Medicine - DIMED, University of Padova, Padua, Italy; 14 Specialty of Child and Adolescent Health, Faculty of Medicine, The University of Sydney, Camperdown, Australia; 15 Department of General Medicine, The Children’s Hospital at Westmead, Westmead, Australia; 16 Division of Pediatric Neurology, Department of Pediatrics, University Hospitals Leuven, Leuven, Belgium; 17 Department of Allergy and Immunology, Children’s Hospital at Westmead, Westmead, Australia

**Keywords:** Complement, C1Q deficiency, interferon, systemic lupus erythematosus, neuroinflammation, Janus-kinase inhibition

## Abstract

Hereditary C1q deficiency (C1QDef) is a rare monogenic disorder leading to defective complement pathway activation and systemic lupus erythematosus (SLE)-like manifestations. The link between impairment of the complement cascade and autoimmunity remains incompletely understood. Here, we assessed type 1 interferon pathway activation in patients with C1QDef. Twelve patients with genetically confirmed C1QDef were recruited through an international collaboration. Clinical, biological and radiological data were collected retrospectively. The expression of a standardized panel of interferon stimulated genes (ISGs) in peripheral blood was measured, and the level of interferon alpha (IFNα) protein in cerebrospinal fluid (CSF) determined using SIMOA technology. Central nervous system (encompassing basal ganglia calcification, encephalitis, vasculitis, chronic pachymeningitis), mucocutaneous and renal involvement were present, respectively, in 10, 11 and 2 of 12 patients, and severe infections recorded in 2/12 patients. Elevated ISG expression was observed in all patients tested (*n* = 10/10), and serum and CSF IFNα elevated in 2/2 patients. Three patients were treated with Janus-kinase inhibitors (JAKi), with variable outcome; one displaying an apparently favourable response in respect of cutaneous and neurological features, and two others experiencing persistent disease despite JAKi therapy. To our knowledge, we report the largest original series of genetically confirmed C1QDef yet described. Additionally, we present a review of all previously described genetically confirmed cases of C1QDef. Overall, individuals with C1QDef demonstrate many characteristics of recognized monogenic interferonopathies: particularly, cutaneous involvement (malar rash, acral vasculitic/papular rash, chilblains), SLE-like disease, basal ganglia calcification, increased expression of ISGs in peripheral blood, and elevated levels of CSF IFNα.

**Supplementary Information:**

The online version contains supplementary material available at 10.1007/s10875-024-01788-5.

## Introduction

Biallelic germline mutations in *C1QA*, *C1QB*, and *C1QC* lead to hereditary complement subunit 1Q deficiency (C1QDef) (OMIM #613,652, #620,321, #620,322). This grouping of very rare inborn errors of immunity has been described to manifest phenotypically as systemic lupus erythematosus (SLE)-like disease (approximately 75% of reported patients fulfilling the 1997 revised ACR classification criteria for definite or possible SLE [1]), with a fraction of patients demonstrating a susceptibility to encapsulated bacteria [2, 3]. C1QDef is thus considered as a cause of monogenic SLE, possibly with more extensive cutaneous involvement, and a lower frequency of anti-dsDNA antibodies and arthritis, than otherwise seen in sporadic cases [1, 3–5]. Clinically differentiating C1QDef from early, apparently sporadic SLE or other monogenic causes of SLE may be challenging. A finding suggestive of C1QDef (or another monogenic defect affecting early components of the complement classical pathway) is normal C3/C4 with unmeasurable lytic activity of the classical complement pathway [6, 7]. Overall, C1QDef is characterised by refractory disease with marked morbidity and increased risk of death.

The molecular pathways leading from a deficit in C1Q subunits to SLE susceptibility have not been fully elucidated, but impaired clearance of apoptotic cells through defective opsonization and downstream complement pathway activation is thought to play a role [8]. More recent data have suggested several alternative mechanisms by which mutations in C1Q components may lead to immune activation. Thus, C1Q may limit auto-reactivity in CD8^+^ T cells by modifying mitochondrial metabolism, as shown recently in a murine model of SLE [9]. Increased interferon α (IFNα) protein production represents another possible link between C1QDef and auto-immunity [4, 10], raising the question as to whether C1QDef might be usefully considered as a monogenic type 1 interferonopathy [11].

In this study we provide a detailed description of 12 cases with genetically proven C1QDef (11 of whom have not been reported previously), noting the diversity and severity of CNS-involvement. We also explore markers of type 1 interferon pathway activation in vivo in a subset of patients, namely expression of interferon stimulated genes (ISGs) in whole blood (*n* = 10), and IFNα protein in cerebrospinal fluid (CSF) (*n* = 2). Further, we describe the effect of hematopoietic stem-cell transplantation (HSCT) and Janus kinase inhibition (JAKi) on the clinical manifestations in five patients (JAKi *n* = 3, HSCT *n* = 2). Finally, we present a review of all previously described genetically confirmed cases of C1QDef.

## Methods

### Patients and Samples

Patients with C1QDef deficiency from the following centres were included: NH SRCC Children’s Hospital (Mumbai), Great North Children’s Hospital (Newcastle upon Tyne), Hospital Necker-Enfants malades (Paris), Children’s Hospital at Westmead (Sydney) and Leuven University Hospital (Leuven). Clinical features, genotype, laboratory and radiological data were provided by the treating physicians in each centre. Patients and/or families gave their informed consent to be included. The clinical and genetic data of one patient (AGS412) were reported previously, in the absence of interferon signalling data [12].

### Transcriptomic Studies

Expression of ISGs in the peripheral blood of patients and controls was assessed using quantitative reverse transcription polymerase chain reaction (qPCR) or a NanoString panel as described previously [13]. These techniques have been shown to provide comparable results [14]. Briefly, blood was collected in PAXgene tubes (PreAnalytix) and, after being kept at room temperature for between 1 and 72 h, was frozen at − 20 °C until extraction. Total RNA was extracted from whole blood using a PAXgene (PreAnalytix) RNA isolation kit. RNA concentration was assessed using a spectrophotometer (FLUOstar Omega, Labtech). qPCR analysis was performed on *n* = 11 samples from 9 patients using the TaqMan Universal PCR Master Mix (Applied Biosystems), and cDNA derived from 40 ng total RNA. Using TaqMan probes for *IFI27* (Hs01086370_m1), *IFI44L* (Hs00199115_m1), *IFIT1* (Hs00356631_g1), *ISG15* (Hs00192713_m1), *RSAD2* (Hs01057264_m1), and *SIGLEC1* (Hs00988063_m1), the relative abundance of each target transcript was normalized to the expression level of *HPRT1* (Hs03929096_g1) and *18S* (Hs999999001_s1). NanoString panel testing was performed on *n* = 3 samples from two patients. For these samples, the copy number of mRNA transcripts of 24 ISGs (including the six listed above), and four housekeeping genes (*ALAS1*, *HPRT1*, *TBP* and *TUBB*), was quantified using a NanoString nCounter™ Digital Analyzer. The raw copy number of mRNA transcripts of each ISG was standardized using the geometric mean of the four housekeeping genes for each individual. The median fold change of the ISGs, when compared to the median of previously collected healthy controls, was used to create an interferon signature (IS) for each individual. ISs from canonical monogenic interferonopathies were used for comparison.

### IFNα Assay

Measurement of IFNα levels in serum and CSF of 2 patients was performed using SIMOA (single molecule array) technology as described previously [15]*.*

### Literature Review

Previously published patients with C1QDef were identified using two approaches: (i) cases described before January 2011 were retrieved from systematic reviews conducted by Schejbel and colleagues [16], and Jlajla and colleagues [6]; (ii) cases published after 2011 were identified through a systematic PubMed search with the term “*C1Q deficiency*” for the period December 2011 to January 2024 All individual publications were reviewed, and patients included where confirmatory genotypes were available, together with at least minimal phenotypic data. We collected data on anti-nuclear antibodies (titre and specificity), CNS, mucocutaneous and renal involvement, and major infections. Data on rarer clinical features were not collected. We defined CNS involvement (non-infectious events i.e. excluding bacterial meningitis), and major infections (mostly septicaemia and meningitis with encapsulated bacteria) as severe events. The term “recurrent infection” was not considered as a category*,* as this term is too vague and frequently reported in patients on immunosuppressive therapy.

### Statistical Tests

Comparisons of reported clinical features and laboratory values were performed using Fischer’s exact test. For pre/post HSCT comparison of ISG expression, the mean expression value of each gene was compared using Friedman test with Dunn’s multiple comparisons. All tests were performed on Graphpad Prism V9.

## Results

### Characteristics of 12 Patients with C1QDef

Genotype, age at symptom onset and therapies used in our cohort are shown in Table 1. Briefly, 10/12 patients displayed biallelic mutations in *C1QA*, one in *C1QB* and one in *C1QC*. Disease onset occurred during the first years of life (median: 18 months, range: 1–72 months). Patients received multiple immune suppressive therapies (*n* = 12/12), fresh-frozen plasma transfusion (*n* = 2/12), plasma-exchange (*n* = 1/12) or stem-cell transplantation (*n* = 2/12). After a median follow-up period of 83.5 months (range 48–168), 4/12 patients had died, and another 7/12 had developed mild to severe neurological sequalae.

**Table 1:** Genotype, age at onset, treatment, outcomes and duration of follow-up of *n* = 12 patients with C1QDef in our cohort

Patient ID	Genotype	Age of onset (months)	Treatments	Morbidity/mortality	Follow-up duration (months)
AGS412	C1QB c.287del G p.Gly96Alafs*50 hom	15	FSDP Aza GC MMF Cyc	Epilepsy Intellectual, visual and motor impairment Good evolution on FSDP and low-dose MMF	168
AGS1000	C1QA c.208C > T p.Gln208* hom	Infancy	HCQ Cyc Aza GC, RTX, IFX HSCT (2x)	Death (Aspergillus pneumoniae shortly after HSCT)	84
AGS1614	C1QA c.79C > T p.Arg27* hom	28	HCQ Cyc Aza GC	Global developmental delay, spasticity, hemiparesis	48
AGS1969	C1QA c.171del T p.Gly58Alafs*224 hom	12	HCQ Aza MMF GC Daps Tofa	Hyper-reflexia Febrile seizures in infancy	100
AGS1970.1	C1QA c.622C > T p.Gln208* hom	18	HCQ Aza MMF GC	Death (due to undefined neurologic event)	100
AGS1970.2	C1QA c.622C > T p.Gln208* hom	43	HCQ MMF MTX GC	Hyperreflexia, clonus	84
AGS2139	C1QA c.622C > T p.Gln208* hom	26	HCQ Aza GC	Spastic paraparesis	52
AGS2522.2	C1QA, c.644 T > A, p.Val215Asp hom	6	HCQ Aza MMF GC PLEX Bari	Death (haemorrhage after renal biopsy during severe flare)	85
AGS3489	C1QC c.205C > T p.Arg69* hom	72	HCQ MMF Cyc GC RTX Bari	Intellectual impairment Epilepsy	83
IND1	C1QA c.606delA p.Gly204Alafs* hom	10	HCQ AzA MMF GC	Spastic diplegia Developmental delay	57
IND2	C1QA c.622C > T p.Gln208* hom	1	HCQ MMF FFP GC	Death (due to undefined neurologic event)	60
AGS3726	C1QA c.127G > A p.Gly43Arg hom	Early infancy	Siro MMF RTX GC HSCT	Alive, no sequalae	60

Treatment column refers to any immunosuppressive treatment received during the disease course. Abbreviations: *Aza* azathioprine, *Bari* baricitinib, *Cyc* cyclophosphamide, *Daps* dapsone, *FFP* fresh-frozen plasma, *FSDP* frozen solvent/detergent-treated plasma, *GC* glucocorticoids, *HCQ* hydroxychloroquine, *het* heterozygous, *hom* homozygous, *HSCT* hematopoietic stem cell transplantation, *IFX* Infliximab, *MMF* mycophenolate mofetil, *PLEX* plasma exchange, *RTX* rituximab. *Tofa* tofacitinib. * Patients V and VI are siblings

**Fig. 1:**
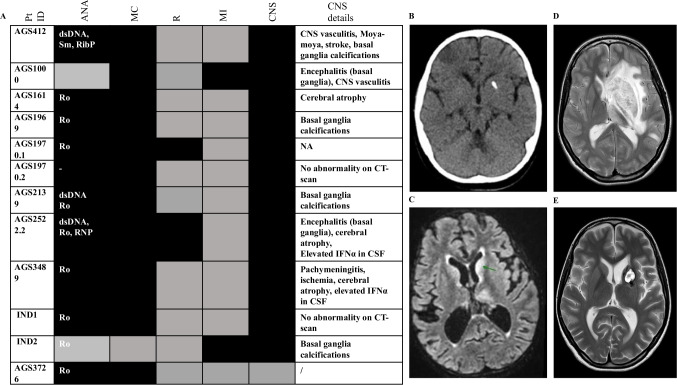
Clinical Features of patients with C1QDef in our cohort. **A** For each patient, presence (dark) or absence (grey) of the following features is indicated: Anti-nuclear antibody (ANA- specific antibodies are written in the cell if present), mucocutaneous (MC), renal (R), major infection (MI) or central nervous system (CNS) involvement. Black and grey boxes indicate, respectively, the presence or absence of disease. Details of CNS involvement are given in the last column. **B **CT-scan of patient AGS412 showing basal ganglia calcification. **C **MRI of patient AGS2522.2 showing encephalitis with signal abnormalities in the basal ganglia and thalami (MRI sequence T2 FLAIR). **D **MRI (T2W) of patient AGS1000 at relapse of CNS inflammation, showing diffuse enlargement of the left basal ganglia, caudate nucleus and thalamus, with mass effect. **E **MRI (T2W) of patient AGS1000 two months after (**D**), showing significant reduction in the size of the lesion and perilesional oedema, and post biopsy changes

Clinical manifestations and the auto-antibody profile of patients in our cohort are shown in Fig. 1A. Most patients demonstrated mucocutaneous manifestations (11/12) such as malar rash, oral ulcers, urticarial, vasculitic or pustular (Sweet’s syndrome) rash and alopecia. CNS involvement was recorded in 11/12, encompassing: basal ganglia calcification, CNS vasculitis, moyamoya disease, encephalitis involving the basal ganglia, cerebral atrophy and pachy-meningitis (Fig. 1B-E). By contrast, renal disease and major infections were rare (2/12). Most patients tested positive for ANA (anti-nuclear antibodies) and anti-Ro antibodies (10/12 and 9/12, respectively).

### Elevated Type 1 Interferon Signature in C1Q Deficiency

**Fig. 2:**
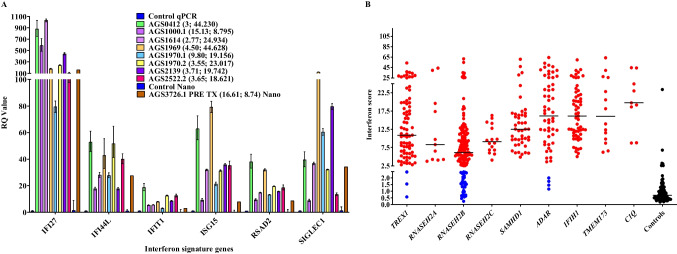
ISG expression in peripheral blood of C1QDef. **A** Expression of 6 ISGs in peripheral blood of *n* = 9 C1QDef patients compared to controls. ISG expression was determined either by qPCR (*n* = 8 patients), or NanoString (*n* = 1 patient). Age at sampling (years) and Interferon score are shown next to each patient ID. **B** Interferon score of *n* = 426 samples from canonical monogenic interferonopathies (results are grouped by mutant genotype), *n* = 79 controls and *n* = 9 C1QDef patients. Black: controls, red: elevated IS, blue: patient with IS in the range of controls. Whiskers show mean ± error of samples analysed using qPCR. Of note, ISG expression data of patient AGS3489 are not shown here (see Methods)

We assessed ISG expression in the whole blood of 10 patients, recording an elevated expression in all patients (Fig. 2A). The interferon signature was found to be in the range of the canonical monogenic interferonopathies Aicardi-Goutières syndrome and STING-associated vasculopathy of infancy (Fig. 2B). In addition, we performed IFNα protein measurement using SIMOA in serum and CSF of two patients with CNS involvement. Both patients (AGS2522.2 and AGS3489, Fig. 1A) displayed elevated serum and CSF IFNα protein levels (223,967.0 and 88,325.8 fg/ml, respectively for patient AGS2522.2; 2468.2 and 159.2 fg/ml, respectively for patient AGS3489) (healthy levels < 10 fg/ml in both serum and CSF).

### Clinical and Biological Effects of HSCT

Two patients underwent HSCT. Because of refractory CNS vasculitis, patient AGS1000 was first transplanted using tissue from a mismatched parent (i.e. carrier of one C1Q pathogenic variant). A low stem cell dose led to associated low donor chimerism and undetectable C1Q serum level, but normalized CH50 assay. Two years after HSCT she presented with a relapse of severe CNS inflammation involving the basal ganglia (Fig. 1D). Brain biopsy showed features of vasculitis (presence of mixed B and T cell perivascular and diffuse infiltrates, and fibrinoid necrosis) (Fig. S1). She was treated with high doses steroids, rituximab and mycophenolate mofetil, leading to marked improvement on MRI (Fig. 1E). A second transplantation using a matched unrelated donor was performed. The patient died shortly thereafter from disseminated fungal infection. The second patient (AGS3726) was transplanted using a matched unrelated donor in the context of severe mucocutaneous disease. At last visit (one month after HSCT), the patient was doing well, with normalized CH50 and C1q levels. Transcriptomic data pre/post HSCT were available for the two patients. HSCT significantly reduced ISG expression to the range of controls (Fig. S2).

### Clinical Effects of JAK Inhibition in Three Patients with C1Q Deficiency

**Fig. 3:**
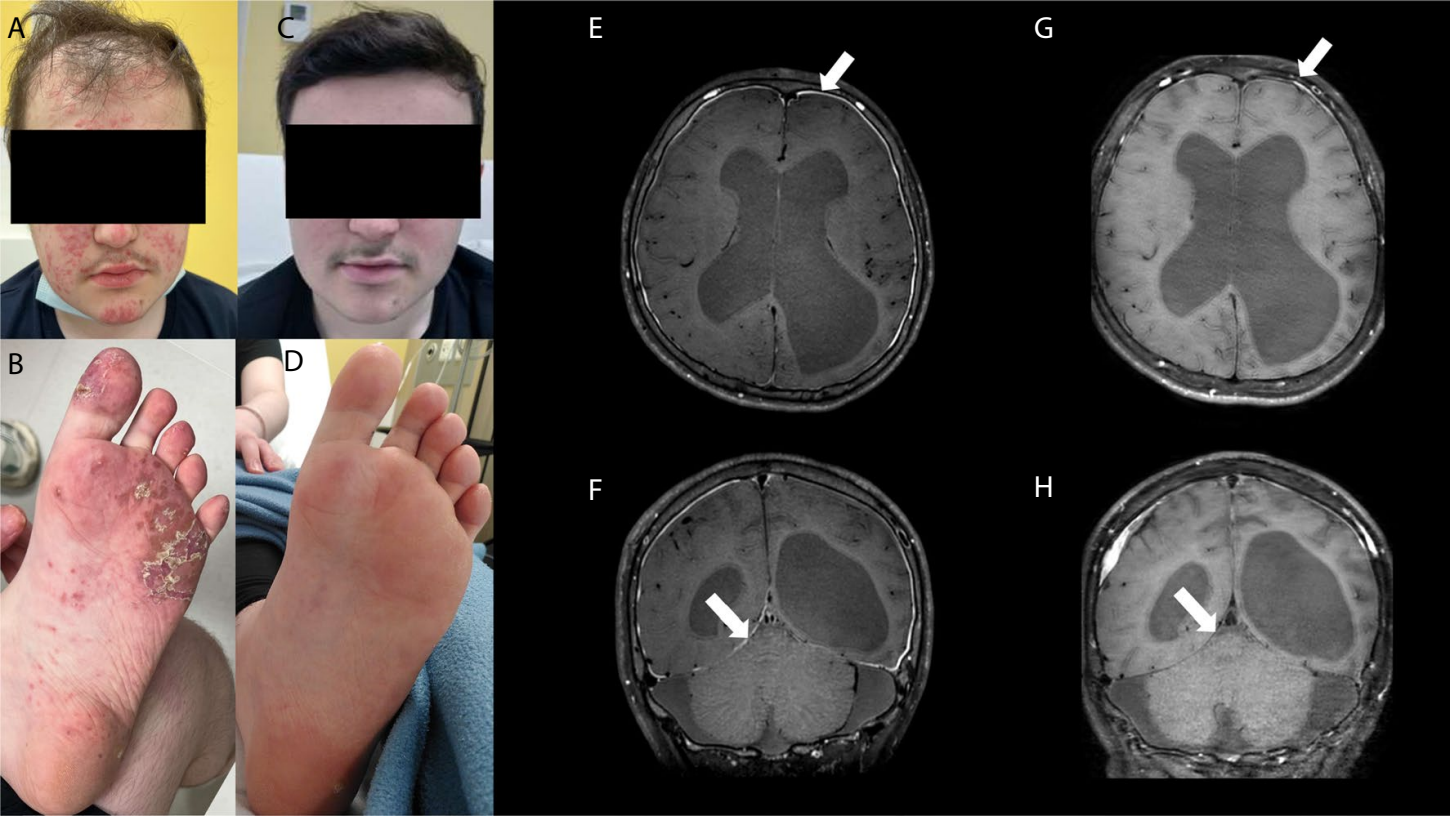
Clinical and radiological effects of JAKi in a patient with C1QDef. **A-D** Clinical picture of the face of patient AGS3489 and foot 2 weeks before (**A-B**) and 9 months after (**C-D**) initiation of treatment with baricitinib. The complete list of therapies at each time point is given in Supplementary Table 2. **E–F** Cerebral MRI (T1 sequence with contrasts) of patient AGS3489 3 months before initiation of baricitinib showing cerebral atrophy and pachymeningitis (arrows indicate meningeal thickening with enhancement). **G-H** Evolution of MRI after 6 months of baricitinib showing improvement in meningeal thickening and enhancement. Of note, a subdural hematoma appeared after lumbar puncture

Three patients in our cohort were treated with JAK-inhibition, with differing outcomes. Patient AGS3489 was started on baricitinib 4 mg/d at age 16 years in the context of active disease despite moderate dose steroids, mycophenolate mofetil and rituximab (see Table S1 for details). Before baricitinib was started, he displayed cutaneous vasculitis, alopecia (Fig. 3A-B), persistent non-infectious pachy-meningitis (Fig. 3E-F), refractory focal epilepsy and elevated expression of ISGs (interferon score = 14.4, normal < 2.7; data not shown in Fig. 2 as performed in another laboratory). After 9 months on baricitinib there was a marked improvement in cutaneous disease **(**Fig. 3C-D), so that steroids could be tapered from > 12 mg methylprednisolone daily to 6 mg daily. Interestingly, therapy also seemed to improve the associated CNS disease, as signs of pachy-meningitis resolved on MRI (Fig. 3G-H) and focal seizures decreased from weekly to three-monthly crises (with concurrent adaptation of anti-epileptic therapy).

Patient AGS2522.2 was started on baricitinib 4 mg/d at age 7 years in the context of refractory membranoproliferative glomerulonephritis despite mycophenolate mofetil, steroids and hydroxychloroquine. Under therapy with baricitinib, steroids and hydroxychloroquine, renal disease worsened and severe diffuse encephalitis developed, requiring plasma-exchanges and cyclophosphamide therapy. The patient died of renal and digestive haemorrhage after a renal biopsy in the context of severe, uncontrolled disease (7 months after baricitinib initiation).

Patient AGS1969 has been treated with tofacitinib 2.5 mg twice daily for two years, with only moderate clinical improvement of skin and minimal CNS involvement (hyperreflexia).

### Characteristics of 77 Previously Described Patients with C1QDef

We reviewed previously reported cases of C1QDef, focusing only on genetically confirmed cases. We identified 66 such patients in total (*n* = 32 from previous reviews, *n* = 34 published cases in 19 publications since 2011). We then combined these data with those of the patients that we report here (*n* = 12 patients including AGS412 who was described by Troedson et al. [12]) (Table S2).

**Fig. 4:**
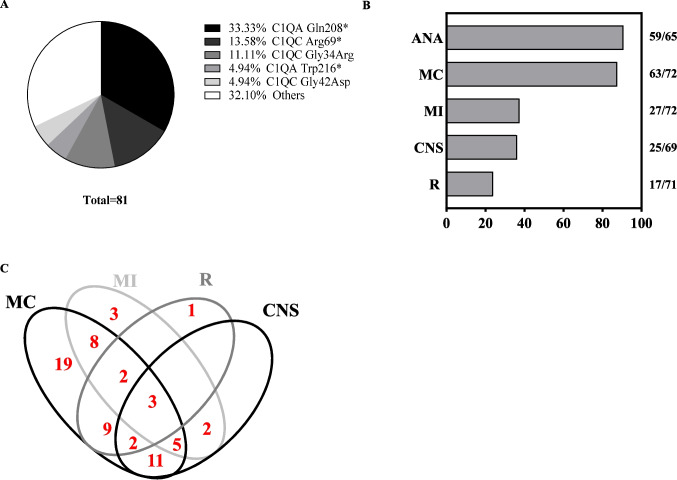
Review of clinical features and genotypes of *n* = 77 published cases with genetically confirmed C1QDef. **A** Most frequent variants reported in C1QDef (73 homozygous and 4 compound heterozygous individuals). **B** Rate of ANA positivity (ANA), mucocutaneous manifestations (MC), major infections (MI), CNS (CNS) and renal involvement in C1QDef. The absolute numbers of cases with data available for each feature is shown on the right. **C** Venn diagram showing the co-occurrence of cardinal features of C1QDEF in *n* = 68 patients with data available for all cardinal features. Only 3 patients were diagnosed without any of these features

Regarding genotype, most patients were homozygous (only 5.2% were compound heterozygous), consistent with a high rate of reported consanguinity. Variants were seen in *C1QA*, *CQ1B*, and *CQ1C* in 55.8%, 15.6%, and 28.6% of the 77 patients, respectively. The most frequent variants are shown in Fig. 4A, which were recorded across different ethnic backgrounds (Table 2, Fig. S3), thereby suggestive of mutational hotspots rather than founder effects.

**Table 2:** Three most frequent variants associated with C1QDef reported in the literature

Gene	Variant	Country of origin/ethnicity in patients with genetically confirmed C1QDEF reported in the literature	GNOMADv4.4—allele frequency	GNOMADv4.4 – Genetic ancestry group
C1QA	Gln208*	India Iran Iraq Turkey Slovakia Cyprus Caucasian	0.00002531	South Asian European (non-Finnish)
C1QC	Gly34Arg	Dutch Caucasian Arabic Indian Pakistani	0.00008212	Middle Eastern European (non-Finnish) Ashkenazi Jewish South Asian Admixed American European (Finnish) Remaining
C1QC	Arg69*	Dutch Kosovo Slovakia	0.000003873	East Asian European (non-Finnish)

See Supplementary Fig. 2 for more information on variant frequency in each ethnic group

Review of all 77 C1QDef cases confirmed a high frequency of mucocutaneous manifestations (present in 87.5% of patients) (Fig. 4B). CNS involvement was reported in 36.2% of patients; cerebral MRI/CT results were described in 17 of those patients, with 9 (52.9%) demonstrating basal ganglia or deep grey matter involvement. Renal disease and major infections were reported in 23.9% and 37.5%, of patients respectively. ANA titres were positive in 90.8% (59/65) of patients. Although techniques have evolved over time and may vary across laboratories, we analysed the most frequent ANA specificities. At least one ANA specificity was reported in 88.1% (52/59) of patients: Ro in 47.5% (28/59), Sm in 42.4% (25/59), RNP in 32.2% (19/59), and native DNA/dsDNA in 18.6% (11/59). The rate of antibody positivity against native DNA/dsDNA was possibly higher in patients with renal involvement than in those without (7/17 = 41.2% *vs* 4/54 = 7.4%, p = 0.0027), although this result should be interpreted with caution since more patients in the non-renal groups had missing ANA specificity data (*n* = 11/54 *vs* 1/17).

We then analysed the co-occurrence of the major clinical manifestations (mucocutaneous / renal / CNS involvement and major infections) in *n* = 68 patients with data for these systems (Fig. 4C), finding the most common phenotypes to be: mucocutaneous involvement without other cardinal features (27.9%), and CNS and mucocutaneous involvement (16.2%).

We noted a change in the reported frequency of some features over time (Fig. S4A). Thus, CNS involvement was reported in 20.7% (6/29) up to 2011, and in 47.6% (20/42) since then, with the reporting of severe infections falling from 45.5% (18/33) to 24.4% (10/41) (Fig. S4B-C). By contrast, rates of reported renal and mucocutaneous involvement remained stable.

## Discussion

To our knowledge, here we report the largest single cohort of genotypically characterised C1QDef patients yet described, and provide evidence for in vivo activation of the type 1 interferon pathway. Thus, in our cohort of 12 individuals, all patients tested displayed high expression of ISGs in peripheral blood (10/10), and elevated serum and CSF levels of IFNα protein were observed in the two patients assessed. Increased CSF IFNα was previously described in a single patient with C1QDef [4]. In possible keeping with an upregulation of type I interferon signalling, our cohort provides further insights into the severe and heterogenous spectrum of CNS disease that can be seen in C1QDef, including basal ganglia calcification, vasculitis, encephalitis and pachy-meningitis. These features differ in severity, localization and frequency from classical neuro-lupus, where typical MRI lesions are small and focal in subcortical and periventricular white matter [17]. As such, the “SLE-like” phenotype commonly attributed to CQ1D seems to apply particularly to mucocutaneous, renal and articular involvement, but not to CNS disease.

Multiple mechanistic studies have linked C1QDef with increased IFNα signalling. In addition to its well-known roles in binding to apoptotic cells and complement cascade-activation, C1q likely also plays a role in immune-tolerance regulation. Thus, C1q binds to the LAIR-1 receptor to suppress dendritic cell activation, dendritic cells being major IFNα producers [18]. In line with this observation, serum from C1QDef patients was shown to inefficiently suppress IFNα production induced by multiple stimuli in PBMCs and dendritic cells, leading to high IFNα levels in the sera of these patients [4, 10].

In addition to our own cohort of 12 patients, we provide a review of all published cases with a confirmed genetic diagnosis of C1QDef (while a recent review of C1QDef included a greater number of patients, ~ 40% of these had not undergone genetic testing [2]). This analysis confirmed a high frequency of mucocutaneous features, sometimes without any other cardinal stigmata. As previously suggested in smaller cohorts, patients with C1QDef display an auto-antibody profile apparently distinct from sporadic SLE, with a low prevalence of anti-dsDNA, and a high prevalence of anti-Ro antibodies [4, 5]. We highlight a modification of reported clinical features over time, which likely results from several factors, including increased availability of vaccination against encapsulated bacteria, the use of systematic antibiotic prophylaxis in C1QDef, and increased access to diagnostic imaging.

There is currently no standard therapy for C1QDef, but several publications report a poor response to most drugs used in sporadic SLE. C1Q supplementation through fresh frozen plasma infusion [19–21], and hematopoietic stem-cell transplantation [22–24], have been reported in a few patients in addition to standard SLE immunosuppressive drugs. The effect of JAKi has been described in two C1 deficient patients (one with C1Q deficiency, one with C1R deficiency), with a beneficial effect on cutaneous manifestations, and downregulation of ISGs [25]. Although our patients received different dosing regiments of JAKi and of co-medications, our report suggests that JAKi may be effective in treating C1QDef-associated mucocutaneous involvement (present in 88.9% of patients in our review). By contrast, our data indicate that JAKi is not beneficial in all patients with C1QDef, as manifestations such as glomerulonephritis did not respond. Given the suggestion of a marked upregulation of type I interferon signalling in C1QDef, and disease overlap with certain well-characterised type I interferonopathies, therapies more directly targeting type I interferon signalling (such as anifrolumab) might be worthwhile considering. We also show resolution of ISG overexpression after HSCT, a therapy reported to be effective in a few C1QDef patients [23, 24]. A worldwide reported experience of HSCT in 17 patients with C1QDef shows an overall survival rate of 70%, with symptom resolution and freedom from immunosuppression in survivors (Gennery, Buso – unpublished data).

Altogether, our data illustrate the severity of C1QDef, which is associated with significant morbidity (in particular, relating to CNS involvement) and increased mortality (4/12 in our cohort) and a variable therapeutic response to JAKi, thereby emphasising the need for further research to better tailor therapy based on patient phenotype and/or genotype.

## Supplementary Information

Below is the link to the electronic supplementary material.

ESM 1 Figure S1A-B. H&E staining of brain biopsy (inflammatory mass involving left basal ganglia) in patient AGS1000. Figure S2. Expression of 6 ISGs in peripheral blood of two C1QDef patients (analysed with qPCR n = 1, or NanoString n = 1) before (plain bar) and after (shaded) HSCT. Notably, the second sample of patient AGS1000 was taken before the second HSCT (low chimerism). Controls for each technique are shown in blue. Whiskers show mean ± error of samples analysed using qPCR. Mean expression value of each gene was compared using Friedman test with Dunn’s multiple comparisons test. Corresponding p-values are shown on the graph Figure S2. Expression of 6 ISGs in peripheral blood of two C1QDef patients (analysed with qPCR n = 1, or NanoString n = 1) before (plain bar) and after (shaded) HSCT. Notably, the second sample of patient AGS1000 was taken before the second HSCT (low chimerism). Controls for each technique are shown in blue. Whiskers show mean ± error of samples analysed using qPCR. Mean expression value of each gene was compared using Friedman test with Dunn’s multiple comparisons test. Corresponding p-values are shown on the graph. Figure S2. Expression of 6 ISGs in peripheral blood of two C1QDef patients (analysed with qPCR n = 1, or NanoString n = 1) before (plain bar) and after (shaded) HSCT. Notably, the second sample of patient AGS1000 was taken before the second HSCT (low chimerism). Controls for each technique are shown in blue. Whiskers show mean ± error of samples analysed using qPCR. Mean expression value of each gene was compared using Friedman test with Dunn’s multiple comparisons test. Corresponding p-values are shown on the graph. Figure S2. Expression of 6 ISGs in peripheral blood of two C1QDef patients (analysed with qPCR *n* = 1, or NanoString *n* = 1) before (plain bar) and after (shaded) HSCT. Notably, the second sample of patient AGS1000 was taken before the second HSCT (low chimerism). Controls for each technique are shown in blue. Whiskers show mean ± error of samples analysed using qPCR. Mean expression value of each gene was compared using Friedman test with Dunn’s multiple comparisons test. Corresponding p-values are shown on the graph. Figure S3: gnomAD v4.4 frequency of top 3 most frequent variants associated with C1QDef, with frequency in specific ethnic groups. Figure S4: A. Evolution of mucocutaneous manifestations (black square), major infections (black circle), CNS (white circle), and renal involvement (white square) according to date of publication. The number of cases is shown after each chronological interval. B-C. Number of patients with (Y) or without (N) CNS involvement (B) and major infections (C) in previous reviews (≤ 2011) and since then (> 2011). p-value of Fischer’s exact test is shown on the graph. (PDF 1.22 MB)

## Data Availability

No datasets were generated or analysed during the current study.
