# Supplementary material for: Hereditary C1q Deficiency is Associated with Type 1 Interferon-Pathway Activation and a High Risk of Central Nervous System Inflammation
Source: J Clin Immunol. 2024 Aug 28;44(8):185. doi: 10.1007/s10875-024-01788-5 (PMC11358312; doi:10.1007/s10875-024-01788-5)
Supplement: Supplementary file 1 — Figure S1A-B. H&E staining of brain biopsy (inflammatory mass involving left basal ganglia) in patient AGS1000. Figure S2. Expression of 6 ISGs in peripheral blood of two C1QDef patients (analysed with qPCR n = 1, or NanoString n = 1) before (plain bar) and after (shaded) HSCT. Notably, the second sample of patient AGS1000 was taken before the second HSCT (low chimerism). Controls for each technique are shown in blue. Whiskers show mean ± error of samples analysed using qPCR. Mean expression value of each gene was compared using Friedman test with Dunn’s multiple comparisons test. Corresponding p-values are shown on the graph Figure S2. Expression of 6 ISGs in peripheral blood of two C1QDef patients (analysed with qPCR n = 1, or NanoString n = 1) before (plain bar) and after (shaded) HSCT. Notably, the second sample of patient AGS1000 was taken before the second HSCT (low chimerism). Controls for each technique are shown in blue. Whiskers show mean ± error of samples analysed using qPCR. Mean expression value of each gene was compared using Friedman test with Dunn’s multiple comparisons test. Corresponding p-values are shown on the graph. Figure S2. Expression of 6 ISGs in peripheral blood of two C1QDef patients (analysed with qPCR n = 1, or NanoString n = 1) before (plain bar) and after (shaded) HSCT. Notably, the second sample of patient AGS1000 was taken before the second HSCT (low chimerism). Controls for each technique are shown in blue. Whiskers show mean ± error of samples analysed using qPCR. Mean expression value of each gene was compared using Friedman test with Dunn’s multiple comparisons test. Corresponding p-values are shown on the graph. Figure S2. Expression of 6 ISGs in peripheral blood of two C1QDef patients (analysed with qPCR n = 1, or NanoString n = 1) before (plain bar) and after (shaded) HSCT. Notably, the second sample of patient AGS1000 was taken before the second HSCT (low chimerism). Controls for each technique are shown in blue. Whiskers s [file 10875_2024_1788_MOESM1_ESM.pdf]

Table S1. Complete list of oral and IV therapies taken at the time of pictures shown in Figure 3.

Drug	Figure 3A-B	Figure 3C-D
Methylprednisolone	Minimum 12mg/d since > 3 months	6mg/d
Methylprednisolone IV	1000mg during 3 days at the start of baricitinib	/
Mycophenolate mofetil	750mg 2x/d	1000mg 2x/d
Rituximab (IV)	1000 mg / 6 months	1000 mg / 6 months
Hydroxychloroquine	250mg/d	250mg/d
Baricitinib	/	4mg/d
Immunoglobulin (IV)	45g/month	45g/month
Sulfamethoxazole / trimethoprim	800/160 mg 3x/week	800/160 mg 3x/week
Oxcarbazepine	600mg 2x/d	/
Valproate	500mg 2x/d	500mg 2x/d
Brivaracetam	/	100mg 2x/d
Acetylsalicylic acid	80mg/d	/
Omeprazole	40mg/d	40mg/d
Calcium and Vitamin D	1000mg/800UI /d	1000mg/800UI /d

Table S2: List of C1Q deficient patients identified in our literature review

Reference	N of patients
**> 2011**	
(1)	2
(2)	1
(3)	1
(4)	2
(5)	5
(6)	2
(7)	1
(8)	1
(9)	1
(10)	1
(11)	3
(12)	1
(13)	1
(14)	2
(15)	1
(16)	1 *
(17)	2
(18)	4
(19)	2
**<2011**	
(20)	5

(21)	1
(22)	4
(23)	1
(24)	1
(25)	1
(26)	3
(27)	2
(28)	2
(29)	3
(30)	1
(31)	5
(32)	1
(33)	2

* Patient included in our cohort as AGS412

References

1. Jlajla H, Sellami MK, Sfar I, Laadhar L, Zerzeri Y, Abdelmoula MS, et al. New C1q mutation in a Tunisian family. Immunobiology. 2014;219(3):241-6.
2. Higuchi Y, Shimizu J, Hatanaka M, Kitano E, Kitamura H, Takada H, et al. The identification of a novel splicing mutation in C1qB in a Japanese family with C1q deficiency: a case report. Pediatr Rheumatol Online J. 2013;11(1):41.
3. Topaloglu R, Taskiran EZ, Tan C, Erman B, Ozaltin F, Sanal O. C1q deficiency: identification of a novel missense mutation and treatment with fresh frozen plasma. Clin Rheumatol. 2012;31(7):1123-6.
4. Bhattad S, Rawat A, Gupta A, Suri D, Garg R, de Boer M, et al. Early Complement Component Deficiency in a Single-Centre Cohort of Pediatric Onset Lupus. J Clin Immunol. 2015;35(8):777-85.
5. Olsson RF, Hagelberg S, Schiller B, Ringden O, Truedsson L, Ahlin A. Allogeneic Hematopoietic Stem Cell Transplantation in the Treatment of Human C1q Deficiency: The Karolinska Experience. Transplantation. 2016;100(6):1356-62.
6. Chaudhary H, Daniel R, Pilania RK, Anjani G, Sharma M, Pandiarajan V, et al. Catastrophes due to missing complements: C1q deficiency lupus with Kikuchi-Fujimoto disease and macrophage activation syndrome. Rheumatology (Oxford). 2020;59(7):1778-80.
7. van Schaarenburg RA, Daha NA, Schonkeren JJ, Nivine Levarht EW, van Gijlswijk-Janssen DJ, Kurreeman FA, et al. Identification of a novel non-coding mutation in C1qB in a Dutch child with C1q deficiency associated with recurrent infections. Immunobiology. 2015;220(3):422-7.
8. Lubbers R, Beaart-van de Voorde LJJ, van Leeuwen K, de Boer M, Gelderman KA, van den Berg MJ, et al. Complex medical history of a patient with a compound heterozygous mutation in C1QC. Lupus. 2019;28(10):1255-60.
9. Bolin K, Eloranta ML, Kozyrev SV, Dahlqvist J, Nilsson B, Knight A, et al. A case of systemic lupus erythematosus with C1q deficiency, increased serum interferon-alpha levels and high serum interferogenic activity. Rheumatology (Oxford). 2019;58(5):918-9.
10. Zoghi S, Ziaee V, Hirschmugl T, Jimenez-Heredia R, Krolo A, Boztug K, et al. Exome sequencing revealed C1Q homozygous mutation in Pediatric Systemic Lupus Erythematosus. Allergol Immunopathol (Madr). 2018;46(6):594-8.
11. Ekinci Z, Ozturk K. Systemic lupus erythematosus with C1q deficiency: treatment with fresh frozen plasma. Lupus. 2018;27(1):134-8.
12. van Schaarenburg RA, Magro-Checa C, Bakker JA, Teng YK, Bajema IM, Huizinga TW, et al. C1q Deficiency and Neuropsychiatric Systemic Lupus Erythematosus. Front Immunol. 2016;7:647.

13. Zecevic M, Minic A, Pasic S, Perovic V, Prohaszka Z. Case Report: Early Onset Systemic Lupus Erythematosus Due to Hereditary C1q Deficiency Treated With Fresh Frozen Plasma. Front Pediatr. 2021;9:756387.
14. Wolf C, Bruck N, Koss S, Griep C, Kirschfink M, Palm-Beden K, et al. Janus kinase inhibition in complement component 1 deficiency. J Allergy Clin Immunol. 2020;146(6):1439-42 e5.
15. Roumenina LT, Sene D, Radanova M, Blouin J, Halbwachs-Mecarelli L, Dragon-Durey MA, et al. Functional complement C1q abnormality leads to impaired immune complexes and apoptotic cell clearance. J Immunol. 2011;187(8):4369-73.
16. Troedson C, Wong M, Dalby-Payne J, Wilson M, Dexter M, Rice GI, et al. Systemic lupus erythematosus due to C1q deficiency with progressive encephalopathy, intracranial calcification and acquired moyamoya cerebral vasculopathy. Lupus. 2013;22(6):639-43.
17. Dasdemir S, Yildiz M, Celebi D, Sahin S, Aliyeva N, Haslak F, et al. Genetic screening of early-onset patients with systemic lupus erythematosus by a targeted next-generation sequencing gene panel. Lupus. 2022;31(3):330-7.
18. Batu ED, Kosukcu C, Taskiran E, Sahin S, Akman S, Sozeri B, et al. Whole Exome Sequencing in Early-onset Systemic Lupus Erythematosus. J Rheumatol. 2018;45(12):1671-9.
19. Namjou B, Keddache M, Fletcher D, Dillon S, Kottyan L, Wiley G, et al. Identification of novel coding mutation in C1qA gene in an African-American pedigree with lupus and C1q deficiency. Lupus. 2012;21(10):1113-8.
20. Schejbel L, Skattum L, Hagelberg S, Ahlin A, Schiller B, Berg S, et al. Molecular basis of hereditary C1q deficiency--revisited: identification of several novel disease-causing mutations. Genes Immun. 2011;12(8):626-34.
21. McAdam RA, Goundis D, Reid KB. A homozygous point mutation results in a stop codon in the C1q B-chain of a C1q-deficient individual. Immunogenetics. 1988;27(4):259-64.
22. Petry F, Hauptmann G, Goetz J, Grosshans E, Loos M. Molecular basis of a new type of C1q-deficiency associated with a non-functional low molecular weight (LMW) C1q: parallels and differences to other known genetic C1q-defects. Immunopharmacology. 1997;38(1-2):189-201.
23. Pickering MC, Macor P, Fish J, Durigutto P, Bossi F, Petry F, et al. Complement C1q and C8beta deficiency in an individual with recurrent bacterial meningitis and adult-onset systemic lupus erythematosus-like illness. Rheumatology (Oxford). 2008;47(10):1588-9.
24. Tsuge I, Kondo Y, Nakajima Y, Nakagawa N, Imai K, Nonoyama S, et al. Hyper IgM syndrome and complement Clq deficiency in an individual with systemic lupus erythematosus-like disease. Clin Exp Rheumatol. 2010;28(4):558-60.
25. Hoppenreijs EP, van Dijken PJ, Kabel PJ, Th Draaisma JM. Hereditary C1q deficiency and secondary Sjogren's syndrome. Ann Rheum Dis. 2004;63(11):1524-5.
26. Petry F, Le DT, Kirschfink M, Loos M. Non-sense and missense mutations in the structural genes of complement component C1q A and C chains are linked with two different types of complete selective C1q deficiencies. J Immunol. 1995;155(10):4734-8.
27. Petry F, Berkel AI, Loos M. Multiple identification of a particular type of hereditary C1q deficiency in the Turkish population: review of the cases and additional genetic and functional analysis. Hum Genet. 1997;100(1):51-6.
28. Topaloglu R, Bakkaloglu A, Slingsby JH, Mihatsch MJ, Pascual M, Norsworthy P, et al. Molecular basis of hereditary C1q deficiency associated with SLE and IgA nephropathy in a Turkish family. Kidney Int. 1996;50(2):635-42.
29. Marquart HV, Schejbel L, Sjoholm A, Martensson U, Nielsen S, Koch A, et al. C1q deficiency in an Inuit family: identification of a new class of C1q disease-causing mutations. Clin Immunol. 2007;124(1):33-40.
30. Gulez N, Genel F, Atlihan F, Gullstrand B, Skattum L, Schejbel L, et al. Homozygosity for a novel mutation in the C1q C chain gene in a Turkish family with hereditary C1q deficiency. J Investig Allergol Clin Immunol. 2010;20(3):255-8.

31. Slingsby JH, Norsworthy P, Pearce G, Vaishnaw AK, Issler H, Morley BJ, et al. Homozygous hereditary C1q deficiency and systemic lupus erythematosus. A new family and the molecular basis of C1q deficiency in three families. Arthritis Rheum. 1996;39(4):663-70.
32. Mehta P, Norsworthy PJ, Hall AE, Kelly SJ, Walport MJ, Botto M, et al. SLE with C1q deficiency treated with fresh frozen plasma: a 10-year experience. Rheumatology (Oxford). 2010;49(4):823-4.
33. Berkel AI, Petry F, Sanal O, Tinaztepe K, Ersoy F, Bakkaloglu A, et al. Development of systemic lupus erythematosus in a patient with selective complete C1q deficiency. Eur J Pediatr. 1997;156(2):113-5.

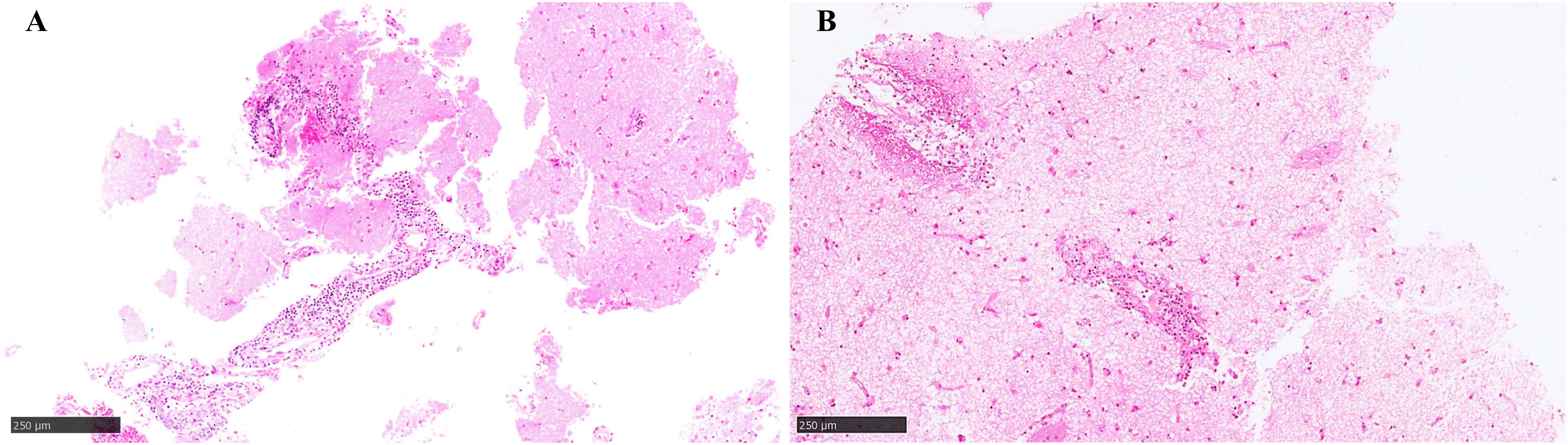
A
250 µm
B
250 µm

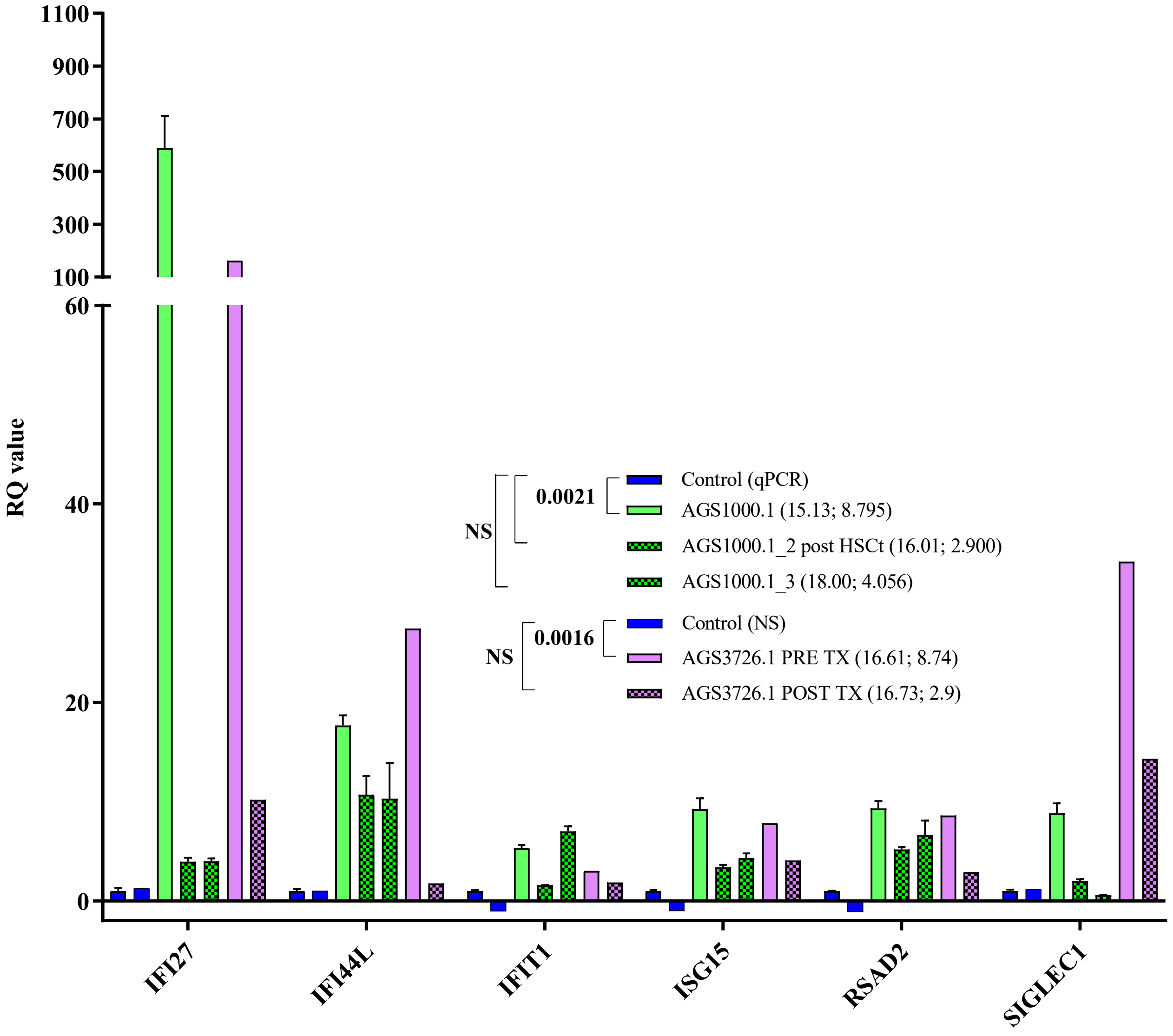

RQ value
1100
900
700
500
300
100
60
40
20
0
NS
0.0021
Control (qPCR)
AGS1000.1 (15.13; 8.795)
AGS1000.1_2 post HSCt (16.01; 2.900)
AGS1000.1_3 (18.00; 4.056)
NS
0.0016
Control (NS)
AGS3726.1 PRE TX (16.61; 8.74)
AGS3726.1 POST TX (16.73; 2.9)
IFI27
IFI44L
IFIT1
ISG15
RSAD2
SIGLEC1

C1QA Gln208*

SNV: 1-22639291-C-T(GRCh38)

Copy variant ID | Gene page | Dataset gnomAD v4.0.0

	Exomes	Genomes	Total
Filters	Pass	No variant	
Allele Count	37		37
Allele Number	1461798		1461798
Allele Frequency	0.00002531		0.00002531
Grpmax Filtering AF (95% confidence)	0.0002899		0.0002899
Number of homozygotes	1		1
Fraction of individuals with >20x coverage	1.0		

External Resources

- dbSNP (rs121909581)
- UCSC
- ClinVar (17073)
- All of Us

Feedback

Report an issue with this variant

Genetic Ancestry Group Frequencies

Genetic Ancestry Group	Allele Count	Allele Number	Number of Homozygotes	Allele Frequency
South Asian	34	86256	1	0.0003942
European (non-Finnish)	3	1111942	0	0.000002698
Remaining	0	60394	0	0.000
Admixed American	0	44722	0	0.000
European (Finnish)	0	53412	0	0.000
Middle Eastern	0	5768	0	0.000
Ashkenazi Jewish	0	26132	0	0.000
East Asian	0	39696	0	0.000
African/African American	0	33476	0	0.000
XX	10	734614	1	0.00001361
XY	27	727184	0	0.00003713
**Total**	**37**	**1461798**	**1**	**0.00002531**

Include: Exomes Genomes

C1QC Arg69*

SNV: 1-22647250-C-T(GRCh38)

Copy variant ID | Gene page | Dataset gnomAD v4.0.0

	Exomes	Genomes	Total
Filters	Pass	Pass	
Allele Count	2	1	3
Allele Number	622544 *	152116	774660 *
Allele Frequency	0.000003213	0.000006574	0.000003873
Grpmax Filtering AF (95% confidence)	9.500e-7	0	8.000e-7
Number of homozygotes	0	0	0
Fraction of individuals with >20x coverage	1.0	1.0	

Warning This variant is covered in fewer than 50% of individuals in gnomAD v4.0.0 exomes. Allele frequency estimates may not be reliable.

External Resources

- dbSNP (rs377549148)
- UCSC
- ClinVar (17070)
- All of Us

Feedback

Report an issue with this variant

Genetic Ancestry Group Frequencies

gnomAD | HGDP | 1KG | Local Ancestry

Genetic Ancestry Group	Allele Count	Allele Number	Number of Homozygotes	Allele Frequency
East Asian	1	41258	0	0.00002424
European (non-Finnish)	2	418042	0	0.000004784
Remaining	0	35140	0	0.000
Admixed American	0	59012	0	0.000
European (Finnish)	0	57654	0	0.000
Middle Eastern	0	4460	0	0.000
South Asian	0	74624	0	0.000
Ashkenazi Jewish	0	24456	0	0.000
African/African American	0	59102	0	0.000
Amish	0	912	0	0.000
XX	1	360676	0	0.000002773
XY	2	413984	0	0.000004831
**Total**	**3**	**774660**	**0**	**0.000003873**

C1QC Gly34Arg

SNV: 1-22644123-G-A(GRCh38)

Copy variant ID | Gene page | Dataset gnomAD v4.0.0

	Exomes	Genomes	Total
Filters	Pass	Pass	
Allele Count	123	7	130
Allele Number	1430776	152192	1582968
Allele Frequency	0.00008597	0.00004599	0.00008212
Grpmax Filtering AF (95% confidence)	0.00008644	0.00003762	0.00008612
Number of homozygotes	0	0	0
Fraction of individuals with >20x coverage	1.0	1.0	

External Resources

- dbSNP (rs200206736)
- UCSC
- ClinVar (17071)
- All of Us

Feedback

Report an issue with this variant

Genetic Ancestry Group Frequencies

gnomAD | HGDP | 1KG | Local Ancestry

Genetic Ancestry Group	Allele Count	Allele Number	Number of Homozygotes	Allele Frequency
Middle Eastern	1	6028	0	0.0001659
European (non-Finnish)	118	1165552	0	0.0001012
Ashkenazi Jewish	2	28882	0	0.00006925
Remaining	4	61456	0	0.00006509
South Asian	3	86230	0	0.00003479
Admixed American	1	53838	0	0.00001857
European (Finnish)	1	61486	0	0.00001626
East Asian	0	43888	0	0.000
African/African American	0	74696	0	0.000
Amish	0	912	0	0.000
XX	53	800262	0	0.00006623
XY	77	782706	0	0.00009838
**Total**	**130**	**1582968**	**0**	**0.00008212**

Include: Exomes Genomes

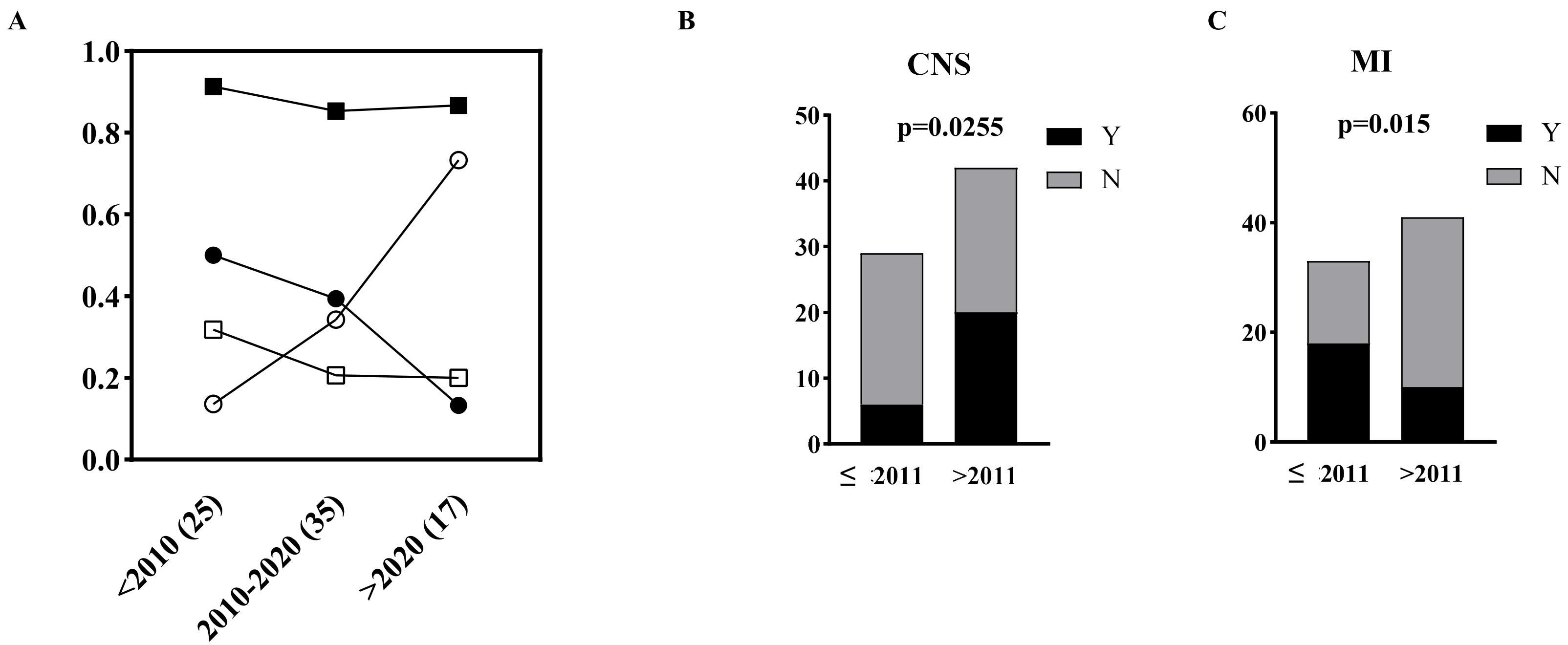
A
1.0
0.8
0.6
0.4
0.2
0.0
<2010 (25)
2010-2020 (35)
>2020 (17)
B
CNS
p=0.0255
Y
N
50
40
30
20
10
0
≤ 2011
>2011
C
MI
p=0.015
Y
N
60
40
20
0
≤ 2011
>2011
